# A novel serum-based medium for drug susceptibility testing of *Mycobacterium tuberculosis*

**DOI:** 10.1186/s12866-026-04946-4

**Published:** 2026-03-13

**Authors:** Kubra Yildirim, Cemilenur Atas, Mehmet Akif Gun, Emel Kaynakci, Ece Simsek, Ahmet Yilmaz Coban

**Affiliations:** 1 Department of Medical Biotechnology, Institute of Health Sciences, Akdeniz University, Antalya, Turkey; 2 Department of Nutrition and Dietetics, Akdeniz University Faculty of Health Sciences, Antalya, Turkey; 3 Faculty of Health Sciences Research Laboratory, Tuberculosis Research Unit, Antalya, Turkey; 4 Medical History and Ethics, Institute of Health Sciences, Akdeniz University, Antalya, Turkey

**Keywords:** Agar proportion method, Sheep serum, *Mycobacterium tuberculosis*, Tuberculosis, Drug susceptbility test

## Abstract

**Background:**

Sheep serum agar (SSA) is a new serum-based medium developed as an alternative to Middlebrook 7H10 agar for the isolation of *Mycobacterium tuberculosis* from clinical samples and for use in antimicrobial susceptibility testing. This study aimed to evaluate the performance of SSA in primary anti-tuberculosis drug susceptibility testing.

**Methods:**

A total of 107 *M. tuberculosis* isolates with different susceptibility profiles were tested simultaneously against primary anti-tuberculosis agents on SSA and Middlebrook 7H10 agar using the proportion method. The findings were compared with those obtained using the BACTEC MGIT 960 (M960) method.

**Results:**

The agreement between the M960 and 7H10 agar proportion method was determined as 93.45% for isoniazid (INH) and ethambutol (EMB), 96.26% for rifampicin (RIF), and 79.43% for streptomycin (STR), respectively. The agreement between the M960 and SSA proportion method was determined as 95.32% for INH, 97.19% for RIF, 92.52% for STR, and 86.91% for EMB, respectively. The agreement between the 7H10 agar proportion method and the SSA proportion method was determined as 98.13% for INH, 99.06% for RIF, 86.91% for STR, and 93.45% for EMB, respectively.

**Conclusions:**

SSA medium was more effective at detecting INH, RIF and STR resistance than 7H10 agar. The results demonstrate the potential of SSA as an alternative medium to 7H10 agar. Further research is needed to confirm its reliability and evaluate its performance.

## Introduction

*Mycobacterium tuberculosis*, a highly successful human pathogen, is the primary cause of tuberculosis (TB), which has the highest morbidity and mortality rates worldwide. This disease, which has managed to survive from approximately 3 million years ago to the present day, continues to affect millions of people every year as one of today’s global health problems. According to the World Health Organization (WHO) 2024 Global TB Report, 10.8 million people were infected with TB globally in 2023 and 1.25 million people died [1, 2].

TB, which has been recorded as one of the deadliest diseases in history, cannot be eradicated today and is also difficult to treat. The main reason for this is drug resistance, which typically arises from spontaneous mutations in the TB bacillus. Of these resistance profiles, multidrug-resistant TB (MDR-TB) and extensively drug-resistant-TB (XDR-TB) profiles is a significant threat to global TB control. Keeping public health safe from these dangerous forms of TB and preventing antibiotic resistance are only possible with the right antibiotics and appropriate treatment regimens [3, 4]. Drug susceptibility testing (DST) is essential to prevent the emergence and spread of antibiotic resistance. Significant advances in laboratory techniques for DST were made in the 1960s, and national and international committees have since developed standardized DST protocols to detect drug resistance. Although conventional methods remain widely used for *M. tuberculosis* in low-income countries, these approaches are often labor-intensive, and time-consumingthey may lack sensitivity, highlighting the need for novel efficient DST media [5, 6]. WHO accepts culture-based phenotypic tests as the “gold standard” methods. The best-known of these methods are the resistance-rate, absolute concentration, and proportion methods. These methods are tested on media such as Löwenstein-Jensen (LJ), Middlebrook 7H9-7H10-7H11 agar at critical concentrations determined for each antibiotic, which vary depending on the medium [7]. The most widely used method is the proportion method. Since the absolute concentration and resistance ratio method has not been sufficiently validated for all anti-TB agents, its use is not recommended. Although culture-based phenotypic DSTs are accepted as the “gold standard” by the WHO, they have limitations, including long incubation periods, time-consuming procedures, strict quality control requirements, and complex laboratory infrastructure [8, 9]. In addition to these methods, new techniques have been developed to rapidly detect drug resistance and shorten the turnaround time [10–16]. Considering the abundance, requirements, and limitations of these defined phenotypic DST methods, the reliability of each method is remarkable. The reliability of DST results is at acceptable levels for isoniazid (INH) and rifampicin (RIF), but does not reach acceptable levels for other anti-TB drugs. Given the heterogeneity of mycobacterial populations, it may be challenging to harmonize methodologies for some antibiotics [17, 18].

Sheep serum agar (SSA) was developed in this study as an alternative to Middlebrook 7H10 agar for the isolation of *M. tuberculosis* from clinical samples and for antimicrobial susceptibility testing. The primary anti-TB drug susceptibilities of 107 *M. tuberculosis* isolates with different susceptibility profiles and together with *M.tuberculosis* H37Rv and H37Ra reference strains were assessed using the proportion method on SSA, a novel serum-based medium. Its minimized chemical composition and enrichment with inactivated sheep serum distinguish SSA from conventional media. In this study, the performance evaluation of SSA was conducted by comparing DST results with those obtained using the proportion method and BACTEC MGIT 960 (M960) on Middlebrook 7H10 agar.

## Methods

### *M.tuberculosis* strains

All strains and isolates tested in the study were obtained from the culture collection of Akdeniz University, Faculty of Health Sciences, Research Laboratory-Tuberculosis Research Unit (Antalya, Türkiye). *M. tuberculosis* H37Rv (ATCC 27294) and H37Ra (ATCC 25177) strains were susceptible to all primary anti-TB drugs and were included as reference strains. A total of 107 *M. tuberculosis* isolates with different resistance profiles were tested. Primary anti-TB drug susceptibilities of all isolates were determined using BACTEC MGIT 960 (M960) (Becton-Dickinson, USA) as the reference method. The resistance profiles of the tested isolates are shown in Table 1.

**Table 1:** Drug susceptibility results of 107 *M. tuberculosis* isolates determined by BACTEC MGIT 960

Susceptibility Profile	Number of Isolates
Susceptible to all primary drugs	34
Resistant to INH	6
Resistant to RIF	2
Resistant to STR	4
Resistant to INH + RIF	8
Resistant to INH + STR	9
Resistant to INH + EMB	1
Resistant to INH + RIF + STR	10
Resistant to INH + RIF + EMB	4
Resistant to INH + STR + EMB	2
Resistant to all primary drugs	27
*Total number*	107

*INH *isoniazid, *RIF* rifampicin, *STR *streptomycin, *EMB* ethambutol

### Preparation of mycobacterial inoculum

All experimental steps were performed in a BSL-3 laboratory, in a class II type B microbiological biosafety cabinet, using personal protective equipment (3 M Versaflo, TR-300), in accordance with the recommended biosafety principles [19]. Bacterial inocula were prepared from fresh cultures grown in Lowenstein-Jensen (LJ) medium, as previously described by Yildirim et al. [20]. Homogenized bacterial suspensions were adjusted to McFarland no 1 turbidity using a McFarland densitometer (BIOSAN Medical-Biological Research & Technologies, Riga, Latvia). The prepared suspension was diluted 10^− 2^ with sterile saline and used as inoculum [9].

### Preparation of antibiotic solutions

In this study, isoniazid (INH), rifampicin (RIF), streptomycin (STR) and ethambutol (EMB) were tested as primary anti-TB drugs. All antibiotics were used in high purity, powder form. Solutions were prepared as recommended by the manufacturer (Sigma-Aldrich, USA). INH, STR, and EMB were dissolved in sterile distilled water, and RIF was dissolved in methanol to prepare stock antibiotic solutions at a concentration of 4096 µg/ml. These stock solutions were used for direct dilution to the single critical concentrations applied in the proportion method. The stock solutions were aliquoted, stored at -80 °C until use [9, 21].

### Preparation of middlebrook 7H10 agar for the proportion method

Middlebrook 7H10 agar (BD Difco^™^) was prepared according to the manufacturer’s recommendations. After being sterilized by autoclaving, it was cooled to 50–55 °C and 10% Middlebrook OADC (oleic acid, albumin, dextrose, catalase) supplement was added. Antibiotic solutions were prepared and added to achieve final concentrations of 0.2 µg/ml for isoniazid (INH), 0.5 µg/ml for rifampicin (RIF), 5.0 µg/ml for ethambutol (EMB), and 2.0 µg/ml for streptomycin (STR). The media were then distributed into 5 ml portions in sterile, screw-capped tubes. Additionally, growth control tubes without antibiotics were also prepared. All tubes were left to solidify at room temperature in a slanted position. The prepared tubes were stored at + 4 °C until the day of the study, with the storage period not exceeding 4 weeks [9, 21].

### Preparation of sheep serum agar (SSA) for the proportion method

A novel serum-based solid medium, termed SSA, was formulated in our laboratory. The medium was prepared using a defined combination of basic chemical constituents, including asparagine, monopotassium phosphate, magnesium salts, agar, and glycerol, which served as the basal nutrient source. After being sterilized by autoclaving, it was cooled to 50–55 °C and 7.5% inactivated, sterile (filtered) sheep serum supplement (Sigma-Aldrich, USA) was added. Antibiotic solutions were added to final concentrations of 0.2 µg/ml for INH, 1.0 µg/ml for RIF, 5.0 µg/ml for EMB, and 2.0 µg/ml for STR. The critical concentrations of antibiotics used in the study were determined in accordance with preliminary validation studies performed on SSA medium. The media were distributed into 5 ml sterile screw-capped tubes. At the same time, growth control tubes without antibiotics were prepared. All tubes were left to solidify at room temperature in a slanted position. The prepared tubes were stored at + 4 °C until the day of the study and the storage period did not exceed 4 weeks [22, 23].

### Implementation of the proportion method

**Fig. 1:**
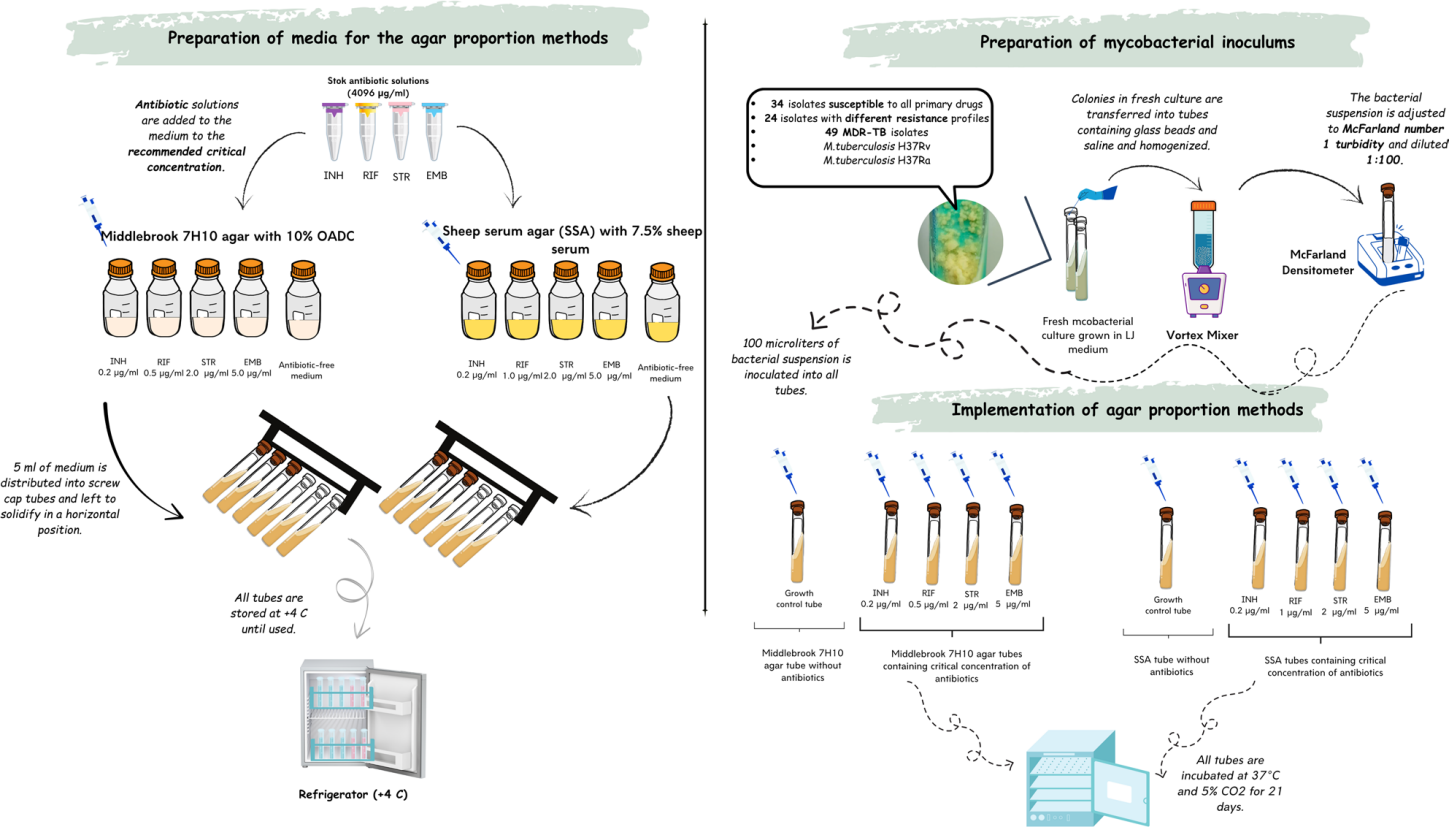
Testing of primary anti-TB drug susceptibilities of 107 *M.tuberculosis* isolates on two different media using the agar proportion method. Demonstration of the applied experimental steps step by step

The proportion method performed on two different media is shown in Fig. 1. Both methods were performed simultaneously using the same mycobacterial inocula. A growth control tube without antibiotics and 4 antibiotic tubes containing critical INH, RIF, STR, EMB concentrations were prepared for all test isolates. These 5-tube experimental sets were prepared separately for 7H10 agar and SSA. Bacterial inocula prepared as described in "Preparation of mycobacterial inoculum" section were inoculated into the growth control tube and test tubes at 100 µl each. After inoculation, all tubes were incubated at 37 °C under 5% CO_2_ conditions for 21 days. The tubes were checked every 3 days for the first week and then weekly. If sufficient growth was observed in the growth control tube at the end of the 21-day incubation, the test was terminated. Drug susceptibility results were evaluated with a 1% ratio approach [9].

### Drug susceptibility testing by M960

Drug susceptibility testing was performed using the fully automated M960 system with the standard SIRE kit, according to the manufacturer’s instructions.

### Statistical analysis

Drug susceptibility test (DST) results were analyzed for specificity, sensitivity, positive predictive value (PPV), negative predictive value (NPV) and agreement, as recommended by the Food and Drug Administration (FDA) [24].

## Results

**Fig. 2:**
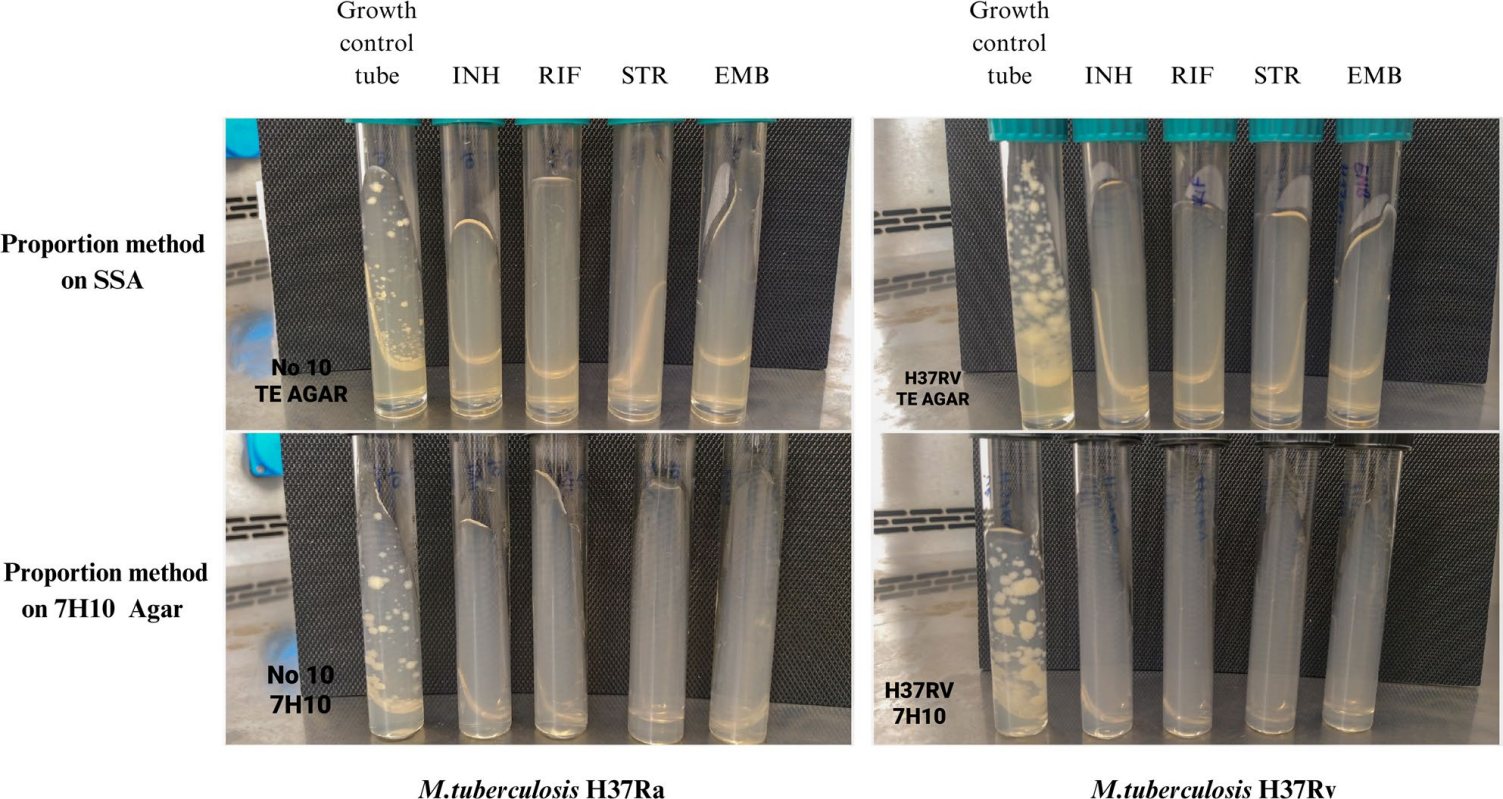
Drug susceptibility results of *M tuberculosis* H37Ra and H37Rv determined by proportion method on 7H10 agar and sheep serum agar (SSA) media. Comparative image of *M. tuberculosis* H37Rv and H37Ra, tested as quality control strains of the study, with the growth control tube and other antibiotic tubes on the 21st day of incubation

On the 21st day of incubation, when sufficient growth was observed in the growth control tubes of all tested isolates, the experiment was terminated and drug susceptibility results were evaluated. *M. tuberculosis* H37Rv and H37Ra, used as quality control strains in the study, were determined to be susceptible to all primary anti-TB drugs in all methods (Fig. 2).

The agreement between the M960 and 7H10 agar proportion method was determined as 93.45% for isoniazid (INH) and ethambutol (EMB), 96.26% for rifampicin (RIF), and 79.43% for streptomycin (STR), respectively (Table 2). The agreement between the M960 and SSA proportion method was determined as 95.32% for INH, 97.19% for RIF, 92.52% for STR, and 86.91% for EMB, respectively (Table 3).

**Table 2:** Comparison of drug susceptibility results of 107 *M. tuberculosis* isolates determined by the proportion method on middlebrook 7H10 agar medium with the results of BACTEC MGIT 960 as a reference method

	Proportion method in 7H10 agar			*Sensitivity (%)*	*Specificity (%)*	*PPV (%)*	*NPV (%)*	*Agreement (%)*
	**BACTEC MGIT 960**	**R**	**S**					
İNH	R	61	6	91,04	97,5	98,38	86,65	93,45
	S	1	39					
RİF	R	49	2	96,07	96,42	96,07	96,42	96,26
	S	2	54					
STR	R	31	21	59,61	98,18	96,87	72	79,43
	S	1	54					
EMB	R	28	6	82,35	98,63	96,55	92,30	93,45
	S	1	72					

*INH* isoniazid, *RIF* rifampicin, *STR* streptomycin, *EMB* ethambutol, *PPV* positive predictive value, *NPV* negative predictive value, *S* Susceptible, *R* Resistance

**Table 3:** Comparison of drug susceptibility results of 107 *M. tuberculosis* isolates determined by the proportion method on sheep serum agar (SSA) with the results of BACTEC MGIT 960 as a reference method

	Proportion method on SSA			*Sensitivity (%)*	*Specificity (%)*	*PPV (%)*	*NPV (%)*	*Agreement (%)*
	**BACTEC MGIT 960**	**R**	**S**					
İNH	R	63	4	94,02	97,5	98,43	90,69	95,32
	S	1	39					
RİF	R	50	1	98,03	96,42	96,15	98,18	97,19
	S	2	54					
STR	R	45	7	86,53	98,18	97,82	88,52	92,52
	S	1	54					
EMB	R	22	13	62,85	98,61	95,65	84,52	86,91
	S	1	71					

*INH* isoniazid, *RIF* rifampicin, *STR* streptomycin, *EMB* ethambutol, *PPV* positive predictive value, *NPV* negative predictive value, *S* Susceptible, *R* Resistance

For INH, the observed discrepancies between the tested methods are summarized below. In the study, 4 *M. M.tuberculosis* isolates were INH-R on M960, whereas they were INH-S on 7H10 agar and SSA. In these 4 isolates, INH resistance could not be detected by either medium in the proportion method. Two isolates were identified as INH-R in the M960 and SSA proportion methods, where as they were identified as INH-S in the proportion method on 7H10 agar. While one isolate was INH-S in M960, it was detected as INH-R in both proportion methods.

For RIF, the observed discrepancies between the tested methods are described below. In the study, 2 *M. tuberculosis* isolates were detected as RIF-S in M960 and RIF-R in the proportion method of both media. One isolate was detected as RIF-R in M960 and RIF-S in the proportion method of both media. One isolate was detected as RIF-R in M960 and SSA with the proportion method, and RIF-S in 7H10 agar with the proportion method.

For STR, the observed discrepancies between the tested methods are detailed below. In the study, 7 *M. tuberculosis* isolates were detected as STR-R in the M960 system, while they were detected as STR-S in both media in the proportion method. *21 M. tuberculosis* isolates were STR-R in the M960 system and were detected as STR-S in the 7H10 agar proportion method. Of these 21 isolates, 14 were detected as STR-R in SSA, in accordance with M960, unlike on 7H10 agar. Only 1 isolate was STR-S in M960 and was resistant in the proportion method of both media.

For EMB, the observed discrepancies between the tested methods are reported below. A total of 7 isolates were detected as EMB-R on M960 and 7H10 agar proportion methods, whereas they were detected as EMB-S in the SSA proportion method. 6 isolates were detected as EMB-R in M960, but EMB resistance could not be detected in either of the two proportion methods. In 1 isolate, while they were detected as EMB-S in M960, they were detected as EMB-R in both proportion methods.

The agreement between the 7H10 agar proportion method and the SSA proportion method was determined as 98.13% for INH, 99.06% for RIF, 86.91% for STR, and 93.45% for EMB, respectively (Table 4).

**Table 4:** Comparison of drug susceptibility results of 107 *M. tuberculosis* isolates determined by the proportion method on sheep serum agar (SSA) with the results of 7H10 agar proportion method as “gold standard”method

	Proportion method on SSA			*Sensitivity (%)*	*Specificity (%)*	*PPV (%)*	*NPV (%)*	*Agreement (%)*
	**Proportion method on 7H10 agar**	**R**	**S**					
İNH	R	62	0	100	95,55	96,87	100	98,13
	S	2	43					
RİF	R	51	0	100	98,21	98,07	100	99,06
	S	1	55					
STR	R	32	0	100	81,33	69,56	100	86,91
	S	14	61					
EMB	R	22	7	75,86	100	100	91,76	93,45
	S	0	78					

*INH* isoniazid, *RIF* rifampicin, *STR* streptomycin, *EMB* ethambutol, *PPV* positive predictive value, *NPV* negative predictive value, *S* Susceptible, *R* Resistance

## Discussion

In this study, the agar proportion method was applied to two media, and the resulting drug-susceptibility test (DST) results were compared with those from BACTEC MGIT 960 (M960) the reference method. The first of these media is Middlebrook 7H10 agar, which is frequently preferred in the agar proportion method. The other is sheep serum agar (SSA), a newly developed serum-based medium whose validation studies have been completed including assessment of the critical antibiotic concentrations for DST applications. This study primarily aimed to evaluate the performance of SSA, developed as an alternative to Middlebrook 7H10 agar for DST applications. In addition, our study also reveals the compatibility/incompatibility between the proportion method and M960 in determining primary anti-TB drug susceptibilities. In this study, 107 *M. tuberculosis* isolates were tested, 34 were susceptible to all primary drugs, 30 had different resistance profiles and 43 were MDR-TB isolates. *M.tuberculosis* H37Rv and H37Ra were included in the study as reference strains. The indirect proportion method was applied using bacterial inocula of the same isolates simultaneously in both media.

When the M960 system was compared with the 7H10 agar proportion method, the agreement rate for INH was 93.45%, when compared with the SSA proportion method, the agreement rate was 95.32%. The agreement rate between the SSA and M960 systems for INH was higher than that with 7H10 agar. Much of the discordance between M960 and the proportion methods arises from the fact that the proportion methods classify isolates that are found to be “resistant” in M960 as “susceptible.” This may be due to INH being tested at different critical concentrations in both methods (0.2 µg/ml for the proportion methods, 0.1 µg/ml for M960) and to the methods being evaluated using different technical procedures.

When the M960 system was compared with the 7H10 agar proportion method, the agreement rate for RIF was 96,26%, and when compared with the SSA proportion method, the agreement rate was 97,19%. Even though there was no significant difference, the agreement rate between SSA and the M960 system for RIF be higher than that with 7H10 agar. In addition, the agreement of the RIFs in both media with the reference method was higher than that of INH.

In a multicenter study by Kontos et al., the proportion method results were compared on M960 and 7H10 agar to determine the primary anti-TB drug susceptibilities of 177 *M. tuberculosis* isolates. For INH, sensitivity was 100%, specificity was 98.6%, and categorical agreement was 98.9%. For RIF, sensitivity was 100%, specificity and categorical agreement was 99.4% [25].

When the M960 system was compared with the 7H10 agar proportion method, the STR agreement rate was 79,43%, and when compared with the SSA proportion method, the agreement rate was 92,52%. As with INH and RIF, the agreement rate between the SSA and M960 systems for STR was higher than that with 7H10 agar. In summary, SSA, a serum-based medium, was more effective at detecting STR resistance among the isolates tested in our study than 7H10 agar. Both media test STR at the same critical concentrations (2 µg/ml). However, the chemical compositions and medium supplements of differ between these two media. SSA uses sheep serum supplementation and contains fewer chemical components than 7H10 agar. In contrast, 7H10 agar uses OADC (oleic acid, albumin, dextrose, catalase) supplementation and has more complex chemical components. These differences may affect the stability, activity, or growth of STR-resistant subpopulations of bacilli in STR-containing medium in different ways. Another factor that may lead to general inconsistency between M960 and both proportion methods is the STR antibiotic’s critical concentration in M960. In the M960 system, STR is tested at 1 µg/ml, whereas in the proportion method it is tested at twice that concentration. This may have made it difficult for the subpopulation with low-level STR resistance to emerge in the proportion method. This can be remedied by re-adjusting and validating the critical concentration of the drug. In 14 isolates detected as STR-R in SSA and M960 but accepted as STR-S in 7H10 agar, colonies ranged from 1 to 5 and could be counted in STR tubes on 7H10 agar on the 21st day of incubation. However, since the proportion method results were evaluated using the 1% ratio principle, these colonies did not affect the sensitivity result. In addition, even if the rate of the STR-resistant subpopulation is minimal, this small number of resistant bacterial communities can appear positive in the M960 system. The success of M960 is due to the technical advantage of the method. Technical differences in the procedures used to measure bacterial growth measurement between the two methods may explain the inconsistency. In principle, fluorometric evaluation is performed when the number of bacilli exhibiting a logarithmic increase over time exceeds a threshold value in the M960 liquid culture system. In this case, even a small number of resistant bacilli willyield a positive result with a logarithmic increase above a certain threshold. Therefore, liquid cultures are considered more sensitive than solid cultures. However, in the proportion method, the number of bacilli inoculated into the solid medium will be equal to the number of colonies formed. In this case, detecting a small number of resistant bacilli that confer low-level resistance in the population will only be possible by increasing their proportion. This is only possible by increasing the inoculum amount of the tested microorganism.

In the study conducted by Said et al., the antibiotic susceptibilities of 343 *M. tuberculosis* isolates to STR, EMB, OFX, and KAN were determined on M960 and 7H11 agar using the proportion method. The agreement between M960 and the proportion method was determined as 61% for STR, 44% for EMB, and 89% for OFX and KAN. In the study where the agar proportion method was used as the gold standard, the sensitivity and specificity of the M960 system were determined as 92% and 37% for EMB, 95% and 37% for STR, 27% and 97% for KAN, and 84% and 90% for OFX, respectively. The low STR agreement rate in this study was even lower than that between 7H10 agar and M960. In this study, the number of isolates detected as STR-R in M960 and STR-S in the proportion method was 124. The success of M960 in detecting STR resistance compared to the proportion method was also observed here. In the same study, the number of isolates detected as EMB-R in M960 and EMB-S in the proportion method was 186 [26].

The discrepancy between the two methods stems from the fact that, as seen in this study, the proportion method yields a “susceptible” result, whereas the M960 method yields a “resistant” result. This situation can be explained by differences in the technical procedures used to evaluate the tests, as described above.

When the M960 system was compared with the 7H10 agar proportion method, the agreement rate for EMB was 93.45%, and when compared with the SSA proportion method, the agreement rate was 86.91%. The agreement rate between the 7H10 agar proportion method and the M960 system for EMB was higher than that for SSA.

The agreement between the 7H10 agar proportion method and the SSA proportion method was determined as 98.13% for INH, 99.06% for RIF, 86.91% for STR, and 93.45% for EMB, respectively. When both proportion methods were compared within themselves, higher rates of agreement were found for INH and RIF (Table 4).

Martilla et al. compared the 7H10 agar proportion method with BACTEC TB-460 and M960 systems for INH, RIF, STR and EMB susceptibility testing of 22 genetically characterized *M. tuberculosis* isolates. The resistant genotype was found to be 87.3% concordant with the 7H10 agar proportion method, 92.7% with BACTEC TB-460, and 96.4% with M960. The results show that M960 has high sensitivity in detecting resistant isolates [27].

It is widely accepted that liquid cultures have higher sensitivity and shorter detection times than solid cultures [28]. This situation is due to technical differences in the procedure used to measure bacterial growth in two different media. Among primary anti-TB drugs, INH and RIF resistance can be detected more reliably and consistently, while STR and EMB resistance are more difficult to detect due to technical limitations [17]. Inconsistencies have been reported for these two antibiotics when comparing liquid and solid media [26, 29–31]. The performance and success of susceptibility tests in determining drug resistance in *M. tuberculosis* vary depending on the drug tested.

The methodology used, whether the “gold standard” or another method considered reliable, does not have the same sensitivity for across all antibiotics tested. As some deficiencies and disadvantages of existing methods are revealed, the need for new methodologies increases [32, 33]. As a result, a conglomerate of methodologies is formed from which laboratories and researchers must choose. Considering the differences in technical procedures, evaluation criteria and application conditions of each, inconsistency between methods is inevitable. Frequent use of drug-susceptible isolates and reference strains in performance/diagnostic evaluations of developed methods helps minimizes inconsistency [34]. However, due to the genetic nature of *M. tuberculosis*, there is heterogeneity in bacillus populations, unlike reference strains. This heterogeneity is an important survival strategy for bacilli. The level of drug resistance in an isolate, defined by the proportion of resistant subpopulations within the overall bacterial population, may be phenotypically expressed as either “susceptible” or “resistant,” depending on the sensitivity of the susceptibility test used [18]. This situation is closely related to the inconsistency between the methods used. Evaluation of susceptibility tests using a large number of clinical isolates with various resistance profiles can produce results closer to reality. In this way, inconsistencies between methodologies can be easily detected and as a result, it can be determined which drugs should or should not be tested using which methodology. DST application may be limited in some environments due to cost and laboratory requirements. However, it is important for determining the appropriate treatment regimen and preventing antibiotic resistance.

These inconsistencies between methods also raise questions about whether appropriate breakpoints are being used. Although inconsistencies between methodologies can be resolved by reorganizing and reviewing the breakpoints for antibiotics, the heterogeneity of bacterial populations may limit this proposal.

In our study, we determined the primary anti-TB drug susceptibilities of 107 *M. tuberculosis* isolates with different susceptibility profiles by applying the proportion method on SSA, a newly developed serum-based medium.

SSA contains fewer chemical components and relies on lower-cost enrichment supplemet compared to Middlebrook 7H10 agar, resulting in a simpler formulation. In addition, SSA does not require specialized automated instrumentation, unlike the MGIT 960 system, which may increase its practical accessibility, particularly in laboratories with limited resources. In this context, the cost comparison among the three tested methods was addressed qualitatively, as a formal cost-effectiveness analysis was not performed. The comparison focused on major cost-determining factors, including consumable requirements and the need for specialized instrumentation. Both SSA and Middlebrook 7H10 agar are solid media that do not require automated systems; however, they differ in the type of enrichment supplements used. The sheep serum–based medium relies on serum as an enrichment supplement, which is generally less costly than proprietary commercial supplements such as OADC required for Middlebrook 7H10 agar. In contrast, the MGIT 960 system depends on proprietary consumables/supplement and dedicated automated instrumentation, and its per-sample cost has been reported in the literature to be approximately 75 USD [35]. In the present study, SSA demonstrated comparable or higher agreement rates than 7H10 agar for the detection of INH, RIF, and STR resistance when compared with the reference method. Together, these results sugget that SSA has the potential to serve as a practical alternative medium for phenotypic DST of *M. tuberculosis*. However, further studies are required to confirm its performance, reliability and cost-related advantages.

## Data Availability

No datasets were generated or analysed during the current study.
